# Exploring the role of neurogenic pathway-linked cholecystokinin release in remote preconditioning-induced cardioprotection^1^

**DOI:** 10.1590/s0102-865020200090000006

**Published:** 2020-10-30

**Authors:** Huilian Li, Cuilan An

**Affiliations:** I BS, Medical Record Room, Central Hospital Affiliated to Shandong First Medical University, Jinan, China. Technical procedures, acquisition and analysis of data.; II BS, Department of Health Care, Central Hospital Affiliated to Shandong First Medical University, Jinan, China. Manuscript preparation and writing.

**Keywords:** Cholecystokinin, Remote Preconditioning, Ischemia, Apoptosis, Neurogenic Pathway, Rats

## Abstract

**Purpose:**

The current study explored the involvement of neurogenic pathway-linked cholecystokinin (CCK) release in RIP-induced cardioprotection in rats.

**Methods:**

Male Wistar rats were subjected to four cycles of alternate episodes of ischemia and reperfusion (five min each) to induce RIP. Thereafter, the hearts were subjected to global ischemia and reperfusion ***ex vivo***. The myocardial damage was assessed by quantifying the levels of heartspecific biochemicals i.e. LDH-1, CK-MB and cTnT. Apoptotic cell injury was assessed by measuring the levels of caspase-3 and Bcl-2. The levels of CCK were measured in the plasma following RIP.

**Results:**

Exposure to RIP significantly increased the plasma levels of CCK and attenuated IR-induced myocardial injury. Administration of CCK antagonist, proglumide significantly attenuated RIP-induced cardioprotection. Administration of hexamethonium, a ganglion blocker, abolished RIP-induced increase in plasma CCK levels and cardioprotective effects. Exogenous delivery of CCK-8 restored the effects of RIP in hexamethonium treated animals.

**Conclusion:**

RIP activates the neurogenic pathway that may increase the plasma levels of CCK, which may act on the heart-localized CCK receptors to produce cardioprotection against I/R injury.

## Introduction

Ischemia-Reperfusion (I/R)-induced myocardial injury is one of the leading causes of mortality and morbidity worldwide^1^. However, there are limited interventions to successfully manage I/R-induced myocardial injury. Remote preconditioning (RIP) is one of the interventions that may have been found to reduce the extent of myocardial injury. In this intervention, exposure of an organ (other than heart) to alternate, short cycles of ischemia and reperfusion confers protection to the heart against sustained I/R injury^2^. The therapeutic utility of RIP has been reported in studies pertaining to animals and humans^3^ ^,^ ^4^. However, the mechanisms involved in RIP-induced cardioprotection are still not fully explored.

Cholecystokinin (CCK) is a neuropeptide that affects growth by regulating appetite. It is synthesized and secreted by enteroendocrine cells in the duodenum^5^. CCK produces actions through CCK-1 and CCK-2 receptors, which are present on different tissues, including heart^6^ ^,^ ^7^. Apart from regulating the local actions in the gastrointestinal tract, studies have documented that this neuropeptide may also produce diverse actions including in the pathophysiology of cancer, behavioral disorders, pain and memory disorders^8^–^10^. CCK also regulates the functions of heart in physiological and pathological conditions^11^–^13^. Considering the presence of CCK receptors on the heart and its role in regulating cardiac functions including ischemia, it was hypothesized that there may be a possible role of CCK in RIP-induced cardioprotection and its release during RIP may be linked to a neural pathway. Therefore, the current investigation explored the involvement of neurogenic pathway-linked cholecystokinin (CCK) release in RIP-induced cardioprotection in rats

## Methods

The experiments were approved by the Central Hospital Affiliated to Shandong First Medical University, under the number: 2020-0618-01. All experiments were performed as per institutional ethical guidelines in the Research Center of the Central Hospital Affiliated to Shandong First Medical University, Jinan, China.

In this investigation, Wistar albino rats of weight 210-240g were used. The doses of CCK-8^14^, Proglumide^15^, heaxmethonium^16^ were selected as per literature reports. The kits for the quantification of lactate dehydrogenase 1 (LDH-1), MB isoform of creatine kinase (CK-MB), cardiac troponins (cTnT), Bcl-2, caspase 3 and CCK were procured from MyBioSource, Inc. San Diego, CA USA.

### Remote ischemic preconditioning (RIP)

After anesthetizing the rats with thiopental sodium (45 mg/kg), a neonatal blood pressure cuff was tied on one of the hind limbs (left). Thereafter, the inflation (up to 150 mm of Hg) and deflation (zero pressure) of cuff was done in an alternate manner to stop (ischemia) and resume (reperfusion) blood supply to the hind limb, respectively. The period of hind limb ischemia and reperfusion constituted five minutes and the hind limb was subjected to four such transient, alternate cycles. After completion of RIP protocol (40 min), the animals were sacrificed and hearts were isolated^17^.

### Ischemia-reperfusion injury

The isolated hearts were mounted on the Langendorff system and perfused with perfused with Kreb's Henseleit solution at 37°C. The inflow of physiological solution was stopped for 30 minutes to induce global ischemia and afterwards, the flow of KH was reinstituted for 120 minutes to establish reperfusion^18^ ^,^ ^19^.

### Assessment of heart-specific biochemicals of heart

In this investigation, IR injury to heart was quantified using three different heart-specific biochemicals i.e. LDH-1, CK-MB, and cTnT. The presence of these biochemicals was assessed in the coronary effluent before subjecting to global ischemia and after instituting reperfusion. To determine the LDH-1 activity, guanidine thiocyanate was added to suppress enzymatic activity of other isoforms of LDH, including LDH-2, LDH-3, LDH-4, and LDH-5. After that, lithium L-lactate and NAD^+^ were added in the sample (containing LDH) to yield pyruvic acid and NADH, and absorbance was measured at 340 nm wavelength.

The determination of CK-MB involved the addition of ADP and creatine kinase in the coronary effluent samples (containing CK-MB) to obtain ATP, which was allowed to react with glucose in the presence of hexokinase. After that, NADP^+^ and glucose-6-phosphate dehydrogenase were added in the reaction mixture that led to the production of NADPH and its absorbance was determined at 340 nm to quantify enzymatic activity.

cTnT was quantified by the sandwich assay-based ELISA kit. The wells of microplate were coated with an antibody specific to cTnT (also called capture antibody). Thereafter, the coronary effluent sample (containing cTnT) was added into the wells that allowed the binding of cTnT to capture antibody. It was followed by the addition of a biotin-conjugated antibody (detection reagent A) specific to cTnT. After washing, avidin conjugated to horseradish peroxidase (HRP) (detection reagent B) was made complex with the biotin-conjugated antibody. After washing, a substrate for HRP i.e. tetramethylbenzidine was added and this yielded the color development, which was measured at 450nm.

### Assessment of apoptotic cell injury

The assessment of apoptotic cell death in the heart was done by quantifying the levels of caspase 3 and bcl-2 in the heart homogenates. These apoptotic parameters were measured using ELISA kits.

### Quantification of plasma CCK levels

The blood was isolated at the time of sacrificing the rats and plasma levels of CCK were measured using ELISA kits.

### Study design

In this investigation, seven groups were employed and eight animals were employed in each group. These groups included (i) I/R injury (ii) RIP (iii) Proglumide (10 mg/kg i.p.) in RIP (iv) Proglumide (20 mg/kg i.p.) in RIP (v) Hexamethonium (20 mg/kg i.p.) in RIP (vi) CCK-8 (20 µg/kg i.v) & hexamethonium in RIP (vii) CCK-8 (20 µg/kg i.v) & hexamethonium in RIP

### Statistics

Mean ± standard deviation (S.D.) was used to represent the data of this study. The results of LDH-1, CK-MB, and cTnT were analyzed using two way repeated measure ANOVA. The data of all other parameters were analyzed using One-way ANOVA. These tests were followed by Tukey's *post hoc* test. The statistical significance was fixed at *p*<0.05.

## Results

### Decrease in I/R-induced myocardial injury in response to RIP

**Figure 1:**
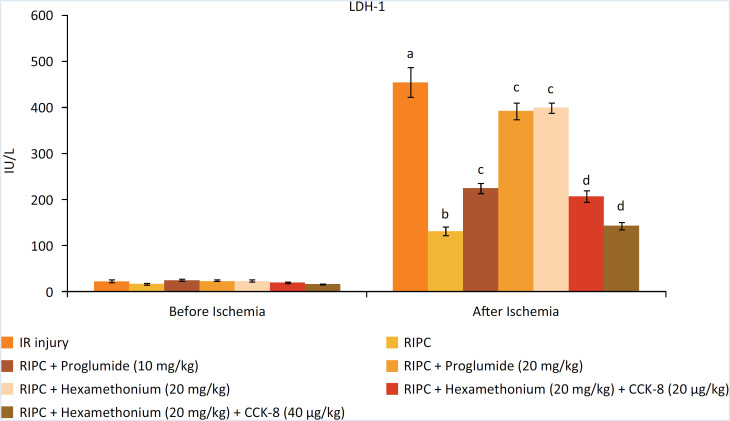
Influence of various treatments on LDH-1 levels. a = *p* < 0.05 *vs*. IR injury, before ischemia; b = *p* < 0.05 *vs*. IR injury, after ischemia; c = *p* < 0.05 *vs*. RIP; d = *p* < 0.05 RIP + hexamethonium.

**Figure 2:**
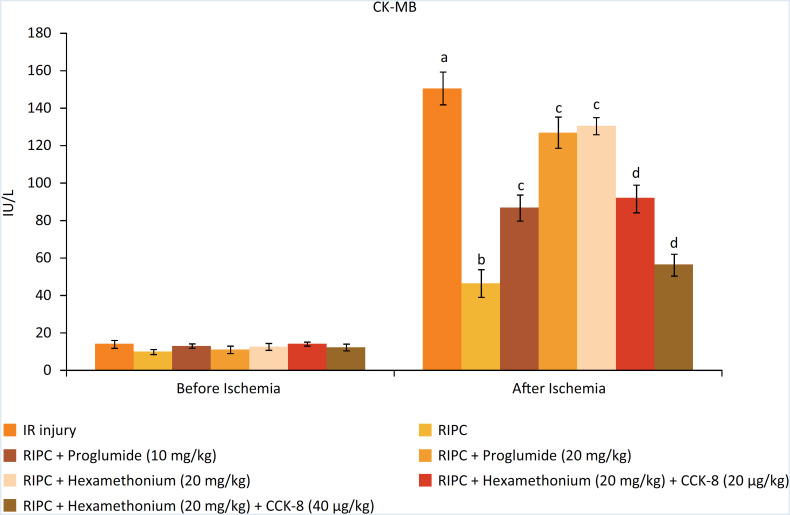
Influence of various treatments on CK-MB levels. a = *p* < 0.05 *vs*. IR injury, before ischemia; b = *p* < 0.05 *vs*. IR injury, after ischemia; c = *p* < 0.05 *vs*. RIP; d = *p* < 0.05 RIP + hexamethonium.

**Figure 3:**
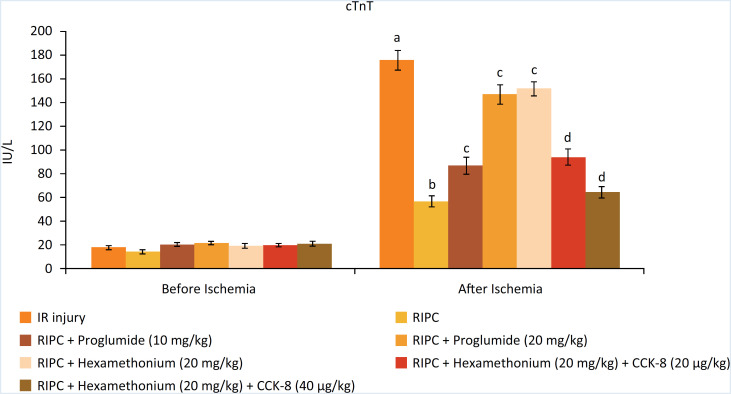
Influence of various treatments on cTnT levels. a= *p* < 0.05 *vs*. IR injury, before ischemia; b = *p* < 0.05 *vs*. IR injury, after ischemia; c = *p* < 0.05 *vs*. RIP; d = *p* < 0.05 RIP + hexamethonium.

**Figure 4:**
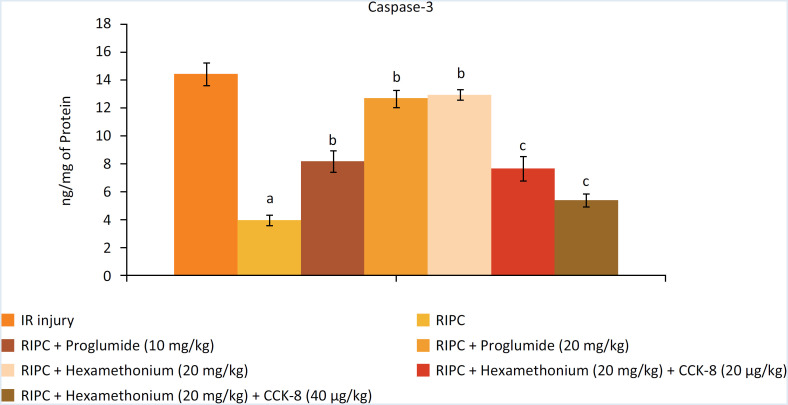
Influence of various treatments on the caspase-3 in the homogenates obtained from heart. a = *p* < 0.05 *vs*. IR injury; b = *p* < 0.05 vs. RIP; c = *p* < 0.05 RIP + hexamethonium.

**Figure 5:**
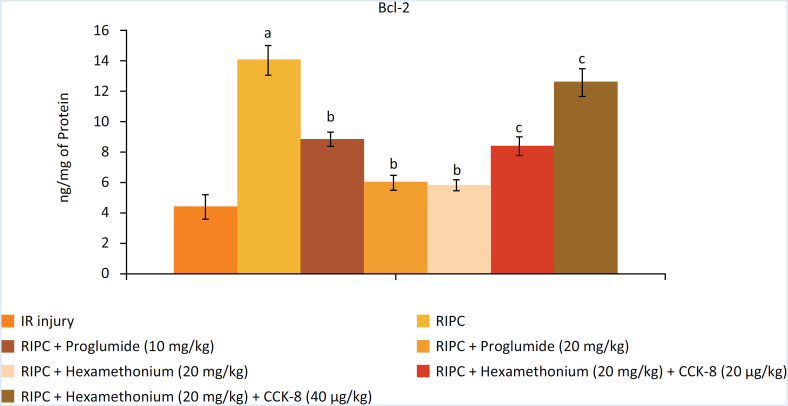
Influence of various treatments on the Bcl-2 in the homogenates obtained from heart. a = *p* < 0.05 *vs*. IR injury; b = *p* < 0.05 *vs*. RIP; c = *p* < 0.05 RIP + hexamethonium.

The levels of heart-specific biochemicals i.e. LDH-1 (Fig. 1), CK-MB (Fig. 2) and cTnT (Fig. 3) were increased in response to ischemia-reperfusion injury. Indeed, their levels were very high during the reperfusion phase as compared to the basal state i.e. before initiating global ischemia to the heart suggesting significant myocardial injury in I/R-subjected rats. Moreover, a marked rise in the parameters of apoptosis i.e. augmentation of caspase-3 (proapoptotic), (Fig. 4) and reduction in Bcl-2 (antiapoptotic) (Fig. 5) was observed in the heart homogenates, suggesting the significant apoptotic cell injury in I/R-subjected rats. However, in RIP-subjected rats, there was a marked decrease in the release of heart-specific biochemicals from the heart following thirty minutes of ischemia (Figs. 1 to 3). Furthermore, RIP decreased the levels of caspase-3 and increased the levels of Bcl-2 suggesting the decrease in apoptotic cell injury.

### Decrease in the cardioprotective effects of RIP in the presence of CCK antagonist and ganglionic blocker

Administration of non-selective CCK antagonist, proglumide (10 and 20 mg/kg) abolished RIP-mediated decrease in heart-specific biochemicals in a dose dependent manner. Moreover, proglumide also abolished RIP-mediated decrease in the parameters of apoptosis in a dose-dependent manner. Administration of ganglionic blocker i.e. hexamethonium (20 mg/kg) also abolished RIP-mediated cardioprotective effects. However, exogenous delivery of CCK-8 (10 and 20 μg/kg i.v) restored the cardioprotective effects of RIP in hexamethonium-administered animals in a dose-dependent manner.

### Increase in the plasma levels of CCK in RIP and its modulation in different experimental groups

A marked rise in the CCK levels was observed in the plasma following RIP protocol. Administration of hexamethonium significantly abolished RIP-mediated increase in the CCK levels. However, exogenous delivery of CCK-8 restored the plasma CCK levels in hexamethonium treated RIP rats. Nevertheless, CCK antagonist did not modulate RIP-mediated increase in CCK levels.

## Discussion

In this current investigation, RIP provided cardioprotection against I/R-induced injury as there was significant reduction in the release of heart-specific biochemicals including LDH-1, CK-MB and cTnT. Moreover, RIP also normalized the parameters of apoptosis in terms of reduction in proapoptotic, caspase-3 and augmentation of antiapoptotic, Bcl-2. This suggests that RIP also attenuated I/R-induced apoptotic cell injury. There have been studies suggesting that RIP attenuates I/R-induced myocardial injury^20^ ^,^ ^21^.

In this current investigation, RIP-induced cardioprotective effects were also associated with a marked increase in CCK levels. CCK is a neuropeptide that helps in regulating appetite and growth. Apart from it, studies have shown its key role in cancer^8^, memory^9^, irritable bowel syndrome^20^, pain^10^ and anxiety^21^. Apart from it, CCK has a key role in regulating cardiovascular functions. Both, CCK1 and CCK2 receptors are expressed on the heart^6^ ^,^ ^7^. Studies have shown that CCK may regulate cardiac function^11^ ^,^ ^12^. Moreover, the marked alterations in the CCK receptors density have been documented in the ischemic heart^13^. Accordingly, it can be hypothesized that RIP augments the levels of CCK in the circulation that travel to the heart to produce cardioprotection against I/R injury. To verify the role of CCK in RIP-induced cardioprotection, non-selective CCK antagonist (CCK1 and CCK2 receptor antagonist) i.e. proglumide was administered prior to RIP stimulus. Administration of proglumide significantly attenuated RIP-induced cardioprotection suggesting that CCK may be critically involved in RIP-induced cardioprotection. To best of our knowledge, it is the first study describing the key role of CCK in RIP-induced cardioprotection against I/R injury.

To further explore the mechanisms involved in RIP-induced increase in CCK release and cardioprotection, hexamethonium (ganglion blocker) was co-administered in RIP-subjected rats. Administration of hexamethonium significantly attenuated RIP-induced increase in CCK release. Moreover, hexamethonium also abolished RIP-induced cardioprotection. There have been studies suggesting that RIP may trigger the activation of neurogenic pathway to induce cardioprotection^22^. Based on the results of this investigation, it seems that RIP may trigger a neurogenic pathway to augment the CCK levels in circulation. In the other words, RIP-induced increase in CCK levels is dependent on the activation of a neurogenic pathway. Exogenous delivery of CCK agonist i.e. CCK-8 significantly restored the cardioprotective effects in hexamethonium-treated RIPC rats, which further suggests the key role of CCK in RIP-induced cardioprotection in I/R-subjected rats. There have been studies documenting the individual role of humoral factors^23^ and the neurogenic pathway^16^ ^,^ ^22^ in RIP-induced cardioprotection. However, the present study attempts to correlate the two different pathways and it may be proposed that the neurogenic and humoral pathways are not totally independent. Rather, these pathways are interlinked and our study proposes that the activation of the neurogenic pathway triggers the release of CCK (as humoral factor), which may act on the heart to produce cardioprotection.

## Conclusion

There is a key role of CCK in RIP-induced cardioprotection in I/R-subjected rats. It may be possible that RIP stimulates the neurogenic pathway leading to increase in the CCK levels, which may act on heart-localized CCK1 and CCK2 receptors to produce cardioprotection against I/R injury.
